# A Social Potential Fields Approach for Self-Deployment and Self-Healing in Hierarchical Mobile Wireless Sensor Networks

**DOI:** 10.3390/s17010120

**Published:** 2017-01-09

**Authors:** Eva González-Parada, Jose Cano-García, Francisco Aguilera, Francisco Sandoval, Cristina Urdiales

**Affiliations:** Departamento de Tecnologia Electronica, ETSI Telecomunicacion, Campus de Teatinos s/n, University of Malaga, Malaga 29010, Spain; gonzalez@uma.es (E.G.-P.); fjaguilera87@gmail.com (F.A.); fsandoval@uma.es (F.S.); acurdiales@uma.es (C.U.)

**Keywords:** mobile sensor networks, WSN, autonomous deployment, SPF, hierarchical routing, geographic routing, self-healing, robots

## Abstract

Autonomous mobile nodes in mobile wireless sensor networks (MWSN) allow self-deployment and self-healing. In both cases, the goals are: (i) to achieve adequate coverage; and (ii) to extend network life. In dynamic environments, nodes may use reactive algorithms so that each node locally decides when and where to move. This paper presents a behavior-based deployment and self-healing algorithm based on the social potential fields algorithm. In the proposed algorithm, nodes are attached to low cost robots to autonomously navigate in the coverage area. The proposed algorithm has been tested in environments with and without obstacles. Our study also analyzes the differences between non-hierarchical and hierarchical routing configurations in terms of network life and coverage.

## 1. Introduction

Wireless sensor networks (WSN) are conformed by a set of interconnected spatially-distributed sensors. These nodes capture information from the environment within their detection range and report to a sink node (SN) that redirects data to the appropriate destination. To achieve this goal, every node needs to be connected to the sink node, directly or through one or more intermediate nodes. The sink node: (i) does not move; (ii) is connected to a main power supply; and (iii) redirects information to an external network. Performance in WSN can be measured in terms of a number of parameters (see Section 3). Specifically, connectivity and throughput depend largely on deployment [1]. Optimal deployment is often not viable, specially if WSN need to cover large, dynamic areas. Instead, WSN often rely on redundant, mesh topologies. However, the deployment of large WSN is still complex [2]. Furthermore, nodes in WSN are prone to failure due to: (i) component malfunctioning; (ii) battery depletion; (iii) environmental factors; and (iv) man-caused factors [3].

Network management techniques include topology management [4]. The main goal of topology management is to achieve sustainable coverage while maintaining connectivity, i.e., to have the largest possible area within the sensing range of at least one sensor and keep at least one communication path between each sensor and the sink node. When sensor failures create holes in the coverage area and/or disconnect full clusters of functional nodes, topology management includes self-diagnostic and self-healing functions. Power control techniques [5] could be applied in such situations to increase the transmission range at the expense of a higher power consumption. Many commercial radio transceivers allow one to adjust the transmission power levels at runtime, but only within a restricted interval, so power control techniques are usually combined with more complex approaches [6]. Some of them are based on strategically adding some redundant nodes so that there is more than one routing path between every pair of sensors in the network (k-vertex connectivity) [7]. The main challenge in these cases is the complexity of planning node deployment using a reduced number of redundant nodes. Alternatively, other methods propose spares for critical nodes. These spares could be passive extra nodes or active nodes nearby the critical ones. In this second case, it is necessary to select as the replacement the node that would cause the least degradation in the network [8].

Some methods work in a reactive way: rather than planning over the full topology, nodes are rearranged according to local criteria, like preserving connectivity or minimizing coverage loss in a given area. In these cases, algorithms can work with as little as one-hop information to detect the failure of critical nodes and decide the best strategy to recover from it [6]. In its simplest implementation, a failed node could be replaced by its nearest uncritical neighbor [9], whereas more complex approaches aim at minimizing movement overhead in order to extend network lifetime [10]. Other proposals deal with routing information to provide short paths between remaining nodes, as well [11]. These techniques are typically used in mobile WSN (MWSN), where nodes have some degree of mobility.

MWSN can autonomously deploy themselves and also adjust their positions if part of the network fails (self-healing) [12]. MWSN are useful for long-term monitorization of large areas and also for emergency deployment of communication networks. In both cases, mobile nodes allow adaptation to changing conditions, including the specifics of the area and also the lifetime of the different nodes. Multiple robot systems (MRS) are adequate for MWSN [3,13,14]. In these systems, robots move to achieve the best possible coverage using all living nodes.

Some approaches for deployment in WSN are based on deliberative algorithms to optimize efficiency [15,16,17,18] and also for self-healing [3,19]. Similarly, self-healing may rely on the deliberative relocation of nodes in the network [20]. Deliberative algorithms reason over a model of the environment. Hence, they tend to be computationally expensive and require information about the problem instance, including network configuration, environment layout, traffic, etc. Instead, reactive deployment assumes that local dispersion leads to global dispersion: hence, each node makes its own decisions based on local factors [21,22,23,24,25]. Reactive deployment is not optimized, so some features, like path redundancy, shortest path to the sink, etc., cannot be guaranteed. However, it is computationally less expensive and, hence, better suited for operation in unknown environments, adaptation to failure and also to dynamic structures where nodes can move.

A popular approach to reactive deployment in MWSN is behavior-based deployment. In behavior-based algorithms, a node relies on several nuclear skills (behaviors). Each behavior associates an input instance to an output action. More complex, emergent behaviors are obtained as the combination of several simpler ones. A node stops moving when the combination of all its behaviors return a null vector. All virtual potential and forces approaches are implementations of behavior-based algorithms [23,24,25]. Alternatively, deployment could follow rules rather than behaviors (e.g., [26]). Rule-based deployment is better fitted to deploy into a given topology, e.g., bus, backbone or ring networks, and also to impose hard constraints, e.g., fix a number of beacons for RSS-based localization [27]. However, behavior-based deployment adapts better to the environment, and it is more adequate for self-healing, especially for multi-node failure, since no fixed node structure needs to be preserved [28].

This work proposes a new behavior-based algorithm for deployment and self-healing of MWSN. The proposed algorithm is a variation of the social potential fields (SPF) [29], originally proposed for navigation in a robot swarm. Force-based strategies have also been proposed for deployment, e.g., [23], or for autonomous repair, e.g., [30]. In one case, forces tend to move nodes away from each other, whereas in the other, healthy nodes tend to move towards the center of the deployment area (assuming it is known). Our proposal is valid for both deployment and self-repair simultaneously, because forces are locally established between nodes, not with respect to specific locations. Each node is affected at all times by a number of forces depending on nearby nodes. Nodes stop when forces are balanced. However, any change that affects this balance will make the node move again. Nodes work uniquely with local information (one-hop). Rather than coping with the state of the network, nodes only care about their own goals, i.e., keep as far from the neighbors as possible and preserve connectivity, plus avoiding collisions, since sensors are mobile, and there might be also other obstacles in the environment. The authors proposed an initial implementation of this SPF-based algorithm for MWSN in [28]. However, the proposed implementation only focused on network expansion and was not adapted to the routing strategy. We propose a new SPF-based algorithm for deployment and self-repair and its adaptation to different routing strategies in Section 2.

The methodology we use to test the algorithm is presented in Section 3, including quality metrics and the test environment. As stated in [3], the cost of mobile sensors is considerably higher than the cost of static ones, and hence, it is generally accepted that deployment of only few of such nodes may be feasible in real environments. Hence, most works with a large number of robot sensors rely on simulations [3,14]. In our work, we have fixed all working parameters of our algorithm using a reduced number of real robots and then tested it with large MWSN in simulation using the Player/Stage environment. Simulations in robotics are not totally reliable because physical agents are affected by a number of factors during operation, but they provide valuable information about how the MWSN would evolve as a whole. Section 4 presents our results, and Section 5 presents conclusions and future work.

## 2. A Behavior-Based Self-Deployment and -Repair Algorithm

### 2.1. An Implementation of SPF for MWSN

This work proposes a behavior-based algorithm valid both for deployment and self-healing. Reactive behaviors are based on the local conditions of the nodes at each time instant. Our algorithm is based on the social potential fields (SPF), originally proposed for swarm robots [29]. SPF assigns simple attraction and repulsion forces to goals and/or constraints. The combination of all forces results in a more complex emergent behavior. In our case, we rely on two repulsion forces and a clustering one. We have purposefully designed two repulsion forces depending on their purpose. One is meant to avoid collision with obstacles and to prevent robots from leaving the area to be covered. The other is meant to expand the network, i.e., to keep the robot as far away from all of the others as possible. Repulsion forces are split into two types, not only because they serve different purposes, but also because it is easier to adjust their parameters when the goal of each one is clearly defined at the reactive level. Forces are active at all times, but nodes only move when they are not balanced. SPF-derived motion is typically smooth and tends to present no discontinuities. In our case, that means that while nodes are moving, there will be a continuous trade-off between keeping away from each other and preserving connectivity.

- Repulsion force(s) $f_{r1}\left(r_{i,j}\right)$ repels the robot from other robots or physical obstacles in its vicinity to prevent collisions. In our implementation, the coverage area boundary is modeled as a virtual obstacle, so that it also repels nodes to prevent them from leaving the area.
- Repulsion force(s) $f_{r2}\left(r_{i,j}\right)$ moves robots away from each other to expand the network. These forces can be calculated using RSS.
- An attraction force (clustering force) $f_{c}\left(r_{i,j}\right)$ increases with $r_{i,j}$ to avoid the loss of communication.

$r_{i,j}$ is the distance between robots *i* and *j*.

Unlike other node relocalization techniques, e.g., [31], our algorithm does not need absolute localization information of elements in the environment to operate. However, it still needs to estimate relative distances between robots and with respect to the coverage area boundaries. The locations of all elements under simulation are always known. In real tests, locations are often estimated by RSS-based trilateration [27,32]. However, RSS is affected by environmental factors and obstacles, so RSS-based localization may yield significant errors. In our case, errors are constrained, because we only need to estimate relative distances between nodes separated by one hop. Besides, in areas where errors could be critical, distances are estimated with an onboard range sensor to prevent collisions with other nodes and obstacles in the environment. Thus, unlike other methods [33], our system does not need to assume that there are no obstacles in the environment, nor have knowledge about its layout. Nevertheless, our system performance would still be affected by localization errors, especially to estimate distances to the deployment area boundaries. Some approaches rely on creating a static beacon infrastructure to reduce localization errors [34]. In open environments where GPS is reliable, some of the network nodes equipped with a GPS receiver may work as beacons to autonomously create this infrastructure during deployment [35]. Mobile robots may statistically combine this information and/or sensor feedback with odometry to improve localization accuracy, e.g., [36]. However, this process may involve computational load not affordable for inexpensive robots. Unlike deliberative navigation systems, reactive approaches do not heavily rely on localization, so RSS-based localization errors are acceptable because they are not accumulative, i.e., they are typically bounded to a few meters. Besides, in our system, nodes typically keep a distance of a few meters between each other, whereas the communication range is larger. Hence, they can use more than three signals for trilateration to achieve some resistance against obstacles in the way and other factors affecting RSS. Localization errors still result in sub-par deployment, especially with respect to the coverage area boundaries, but in reactive approaches, the results are never optimal. However, in order to avoid collisions due to these localization errors and also with obstacles that are not transmitting (robots out of battery, walls, other obstacles in the environment, etc.), robots include a frontal range sensor that is used to estimate repulsion force $f_{r1}$.

Each node is affected by a number of forces depending on its location and all nearby nodes, which are combined to obtain an emergent motion vector. Globally, robots spread over the coverage area, avoiding each other and preserving connectivity. A node stops moving when its emergent motion vector is under a threshold $f_{u}$. If any force affecting the node changes, it may move again. For example, when a node fails, all of its close neighbors stop receiving its signal, i.e., they lose a component of $f_{r2}$. Hence, they must move until they balance all remaining forces. Deployment/self-healing finishes when all nodes stop moving.

### 2.2. Role Definition for Different Routing Mechanisms

Deployment/self-healing algorithms return different network topologies depending on the employed routing algorithms. WSN support different routing mechanisms [37,38]. However, when deployment is not planned and signaling traffic needs to be very limited to extend the network life as much as possible, the most popular routing mechanisms are (greedy) geographic and hierarchical routing. Geographic routing works over multi-hop topologies. Its main advantages with respect to other WSN strategies are [3,38]: (i) it is a stateless, i.e., highly energy efficient, routing strategy; (ii) it adapts quickly to network topological changes; and (iii) it provides good scalability. One of the simplest approaches to geographic routing is the closest neighbor routing: a given node *i* sends a packet to all of its neighbor nodes within its transmission range. However, receiving neighbor nodes only retransmit a message if they are closer to the destination node than the one from which they received the message. A node never retransmits the same packet more than once. This algorithm provokes a partial flooding; but, it is very simple, and it does not require additional signaling for routing.

**Figure 1:**
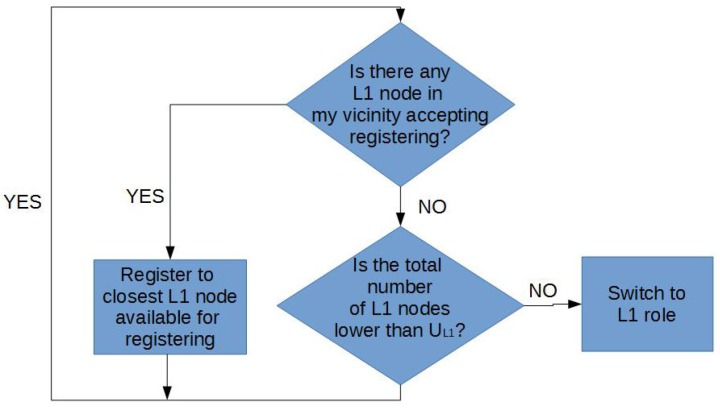
Role definition algorithm for Level 0 (L0) nodes.

Hierarchical routing is a popular approach to point-to-point routing with a very small routing state [38]. Hierarchical routing infrastructure can be autonomously bootstrapped and maintained by the nodes [39]. In its simplest formulation, a hierarchical network includes three node levels. The sink node is a Level 2 node. All nodes connected to the sink node are Level 1 nodes (L1). Finally, nodes connected to a Level 1 node are Level 0 nodes (L0). L0 nodes are registered with a specific L1 node. L1 nodes are registered with the sink node. Typically, an L0 node only exchanges data with the L1 node it is registered with, and that L1 node redirects data to the sink node. The main drawback of hierarchical routing in WSN is that failure in L1 nodes (critical nodes) may lead to loss of all L0 nodes that were registered with them. This problem can be partially mitigated if L0 nodes may become L1 nodes to replace dead ones. Furthermore, in MWSN, nodes may relocate to adapt to this new configuration. In this work, we propose a very simple role-swapping algorithm. Originally, all nodes except the sink start as L0. An L0 node is promoted to L1 if: (i) there is no L1 node in its vicinity; and (ii) the number of L1 nodes for the whole network is under a prefixed threshold that depends on the total number of nodes N. Alternatively, if an L0 node finds in its vicinity an L1 node that still accepts connections, it registers with that node. If an L0 node moves away from the L1 node it is registered with, it can unregister and find a closer one with which to register. This algorithm is presented in Figure 1. It needs to be noted that role swapping occurs when nodes move (deployment or self-healing).

The main difference between both presented routing algorithms is that in geographic routing, all nodes have the same role, whereas in hierarchical routing, nodes may have different roles. The proposed SPF-based algorithm may support both routing strategies if we define node roles, as well. Forces defined in Section 2.1 that affect a node may change depending on the node role:

- $f_{r1}\left(r_{i,j}\right)$ is the same for non-hierarchical and hierarchical topologies, i.e., in both cases, nodes need to avoid obstacles and remain in the coverage area.
- Repulsion force(s) $f_{r2}\left(r_{i,j}\right)$ depends on node roles. In hierarchical routing, L0 nodes are less repelled from L1 nodes than from other L0 nodes or the sink node. Similarly, L1 nodes are less repelled from the sink than from other nodes.
- The clustering force $f_{c}\left(r_{i,j}\right)$: in non-hierarchical networks, nodes are attracted to the sink node: in hierarchical networks, L0 nodes are attracted to L1 nodes, and L1 nodes are attracted to the sink node.

It must be noted that this work does not focus on routing, but on the effect of both routing strategies in deployment topologies. Hence, no routing optimization, nor deep analysis is performed.

**Figure 2:**
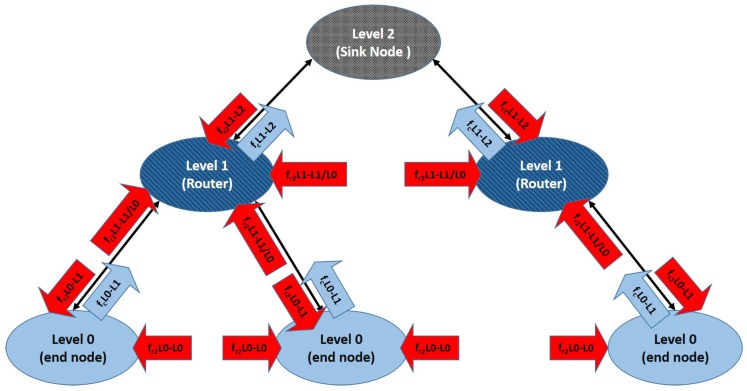
Forces involved in hierarchical network deployment and self-healing.

It can be observed that in geographical routing, all nodes (except the sink) are affected by the same forces. Figure 2 shows forces affecting nodes depending on their roles for hierarchical routing depending on the node level. Attraction and repulsion forces are represented in different colors. The next subsection provides further detail on these forces and how they are adjusted in our system.

### 2.3. Algorithm Implementation

Force parameters need to be heuristically set depending on the physical nature of nodes (robot and RF chipset). Although tests in large networks have been performed under simulation, we have built a small number of real robots to obtain these parameters (see Section 3.2). Table 1 shows the force equations and their respective parameters for both routing mechanisms in Section 2.2. As commented, in geographic routing, all nodes are affected by the same forces. In hierarchical routing, there are different clustering ($f_{c}$) and repulsion forces ($f_{r2}$) between nodes at Levels 0 (L0), 1 (L1) and 2 (sink node, SN).

In order to set the parameters, the following considerations were taken into account. Our robots are built on a Hexbug Spider frame (5.1 inches × 4.3 inches × 4.3 inches), and they are attached with a Texas Instruments EZ430-RF2500, which has been configured to achieve a communication range of 25 m. They also include a SHARP IR range sensor for collision avoidance. Parameters are set so that we can cover approximately an area of 150 m × 150 m with 100 robots.

- Repulsion forces $f_{r1}\left(r_{i,j}\right)$ in our tests are fixed so that robots are not affected by objects farther than 1 m. Obstacles may include static objects and other robots and also the borderline of the area to be covered by the MWSN.
- Repulsion forces $f_{r2}\left(r_{i,j}\right)$ are adjusted to keep at least 2 m between any two robots.
- Clustering forces $f_{c}\left(r_{i,j}\right)$ are adjusted to start affecting robots when they are at least 1.5 m away.

We set the parameters of each force heuristically, using a limited number of robots until their behavior consistently met our requirements, e.g., robots affected only by $f_{r1}\left(r_{i,j}\right)$ did not collide with each other, nor paid attention to obstacles further than 1 m. Obviously, if the size of the robots or the communication range changed, parameters would need to be recalculated using the new physical nodes. After the parameters of all forces were fixed in an isolated manner, all forces were combined into a single emergent motion vector, as proposed in the SPF framework.

**Figure 3:**
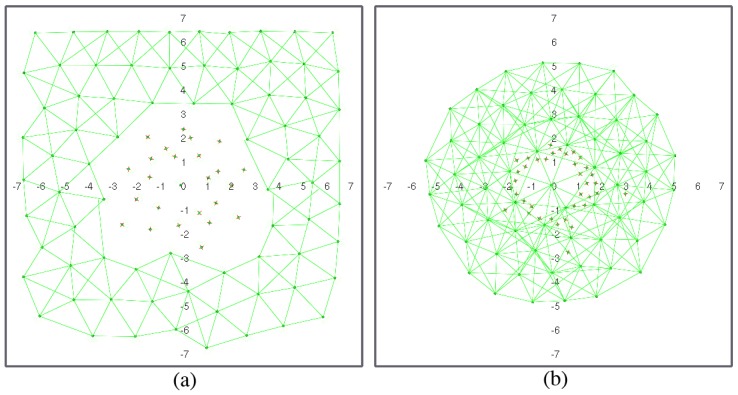
MWSN with geographic routing: (**a**) without clustering force; (**b**) with clustering force (figures are scaled 1:10 m).

**Figure 4:**
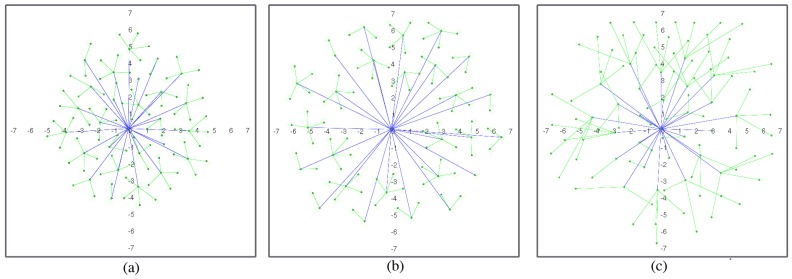
Hierarchical MWSN: (**a**) with all clustering forces; (**b**) without clustering force L1 vs. the sink node (L2); (**c**) without clustering force L0 vs. L1 (figures are scaled 1:10 m).

Repulsion forces are in charge of (i) collision avoidance (including deployment area boundaries) and (ii) spreading the network over the coverage area, so removing $f_{r1}\left(r_{i,j}\right)$ would result in collisions and escaped nodes, and removing $f_{r2}\left(r_{i,j}\right)$ would result in non-deployment. Figure 3 and Figure 4 illustrate the effect of removing clustering forces for both routing strategies. In geographic routing, no clustering force is required for initial deployment as long as the number of nodes is enough to cover the full coverage area. However, when nodes start to die, some node clusters might get disconnected from the sink node. Nodes close to the sink node usually have a higher traffic load and fail first, whereas nodes on the boundaries may have a longer life [19]. However, if they are not compelled to move closer to the sink node after part of the network has failed, they might get disconnected from the rest of the MWSN. Figure 3a shows the same MWSN after 25% of its nodes have died in absence of a clustering force. If a clustering force is added, nodes tend to move closer to the sink node (Figure 3b). They still need to avoid dead nodes in the way, and coverage obviously decreases; but, all living nodes remain connected to the sink node. In a hierarchical network, clustering forces depend on node roles (Figure 2). The force in charge of collision avoidance $f_{r1}$ is the same as in the previous case. However, there are different repulsion forces for deployment in this case. L1 nodes tend to keep away from all of the other nodes, including the sink. However, repulsion from the sink node is stronger, meaning that they are not affected by far L0/L1 nodes. L0 nodes are not repelled by the sink, only by L1 nodes and other L0 nodes. Repulsion from L1 nodes is stronger in this case, meaning that L0 nodes registered to the same L1 node can conform small clusters, but still aim to deploy themselves as spread out as possible. In this case, there are only clustering forces between L1 nodes and the sink and between L0 nodes and L1 nodes. This forces are also in charge of self-healing after role swapping: if a given L1 node fails, one of the nearby L0 nodes will become L1 (Figure 1). At this point, it will start to become strongly attracted to the sink node and, hence, reach a location where it is directly connected to the sink. Besides, it will start to attract L0 nodes. Orphaned nodes or any other L0 node closer to the new L1 node than to its own will try to register to it, and hence, one-hop connectivity to the sink will be restored.

Figure 4a shows an example of an MWSN deployed with all clustering forces in Table 1. If the clustering force between L1 nodes and the sink node is removed, L1 nodes tend to travel further from the sink node (Figure 4b), sometimes even further than their own L0 nodes. If the clustering force between L0 and L1 nodes is removed, deployment is less homogeneous, and the distances from L0 to L1 nodes are more random (Figure 4c).

## 3. Methodology

The proposed algorithm is going to be tested for deployment and self-healing both for geographic and hierarchical routing configurations. This section describes our methodology.

### 3.1. Evaluation Parameters

Deployment and self-healing algorithms in an MWSN can be evaluated in terms of coverage and network life. These two factors are going to be analyzed in terms of the following parameters, which we originally proposed in [28]:

- Blanket coverage: percentage of the deployment region *A* covered by at least one sensor. Coverage *C* is the ratio between the union ∪ of all $A_{i}$ and *A*, $A_{i}$ being the round area covered by node *i*. For *N* sensors:
  (1) $$C=\frac{\cup_{i=1\ldots N}A_{i}}{A}$$
  If we assume that cell *i* has a probability $p_{i}$ of detecting an event on the cell, we can model Equation (1) with a probabilistic grid of *M* cells [40]. Any event at cell *i* can be detected by several nodes, i.e., node *j* may detect an event at cell *i* with a probability $p_{ij}$. Hence, $p_{i}$ can be calculated from the probability of an event going undetected at cell *i* (${\bar{p}}_{i}$):
  (2) $$p_{i}=1-{\bar{p}}_{i}=1-\prod_{N}\left(1-p_{ij}\right)$$
  Finally, coverage *C* is obtained using Equation (3):
  (3) $$C=\sum_{i=1}^{M}\frac{p_{i}}{M}$$
- Energetic efficiency: The cost of deployment and self-healing depend on distance *d* traveled by a node to its current location; and time *t* to reach its current location [41].
- Energy cost when nodes are not moving depends on the uniformity *U* of the deployment topology. In a network of *N* nodes:
  (4) $$U=\frac{1}{N}\sum_{i=1}U_{i}$$
  (5) $$U_{i}={\left(\frac{1}{K_{i}}\sum_{j=1}^{K_{i}}{\left(r_{i,j}-{\bar{r}}_{i}\right)}^{2}\right)}^{1/2}$$
  $K_{i,j}$ being the number of neighbors of node *i*, $r_{i,j}$ being the distance between nodes *i* and *j* and ${\bar{r}}_{i}$ being the average distance between node *i* and its neighbors.
- The average power that nodes require to send a message to the network $\bar{P}$:
  (6) $$\bar{P}=\frac{1}{N}\sum_{i=1}^{N}{\bar{P}}_{i}$$
  ${\bar{P}}_{i}$ being the average power that node *i* needs to send a message to the network. ${\bar{P}}_{i}$ has an impact on the network lifetime, and it can be obtained as:
  (7) $${\bar{P}}_{i}=\frac{1}{N}\sum_{i=1}^{N-1}P_{ij}$$
  $P_{ij}$ being the power needed to send a message from node *i* to *j*. This power depends on the physical features of the RF chipset the network is using.
  If messages need to hop through *k* nodes, it is necessary to add the involved transmission power between each of the two nodes:
  (8) $$P_{ij}=P_{i1}+\ldots +P_{ik}$$
- Networks are unbalanced when some nodes consistently transmit more packets than others. In the non-balanced situation, the life time of loaded nodes is significantly shorter that the rest. Failure in some critical nodes may lead to disconnection of large areas of the network. Unbalance can be analyzed by the evaluation deviation in the number of routed packets per sent message in the network ($\sigma Msg_{Tx}$). If $\sigma Msg_{Tx}$ is high, some nodes are routing far more traffic than the rest.

### 3.2. Work Environment

**Figure 5:**
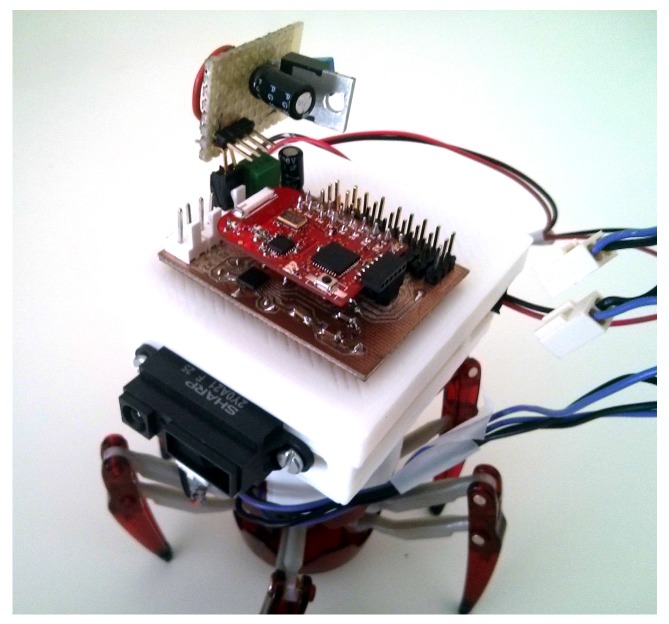
Node hardware structure.

**Figure 6:**
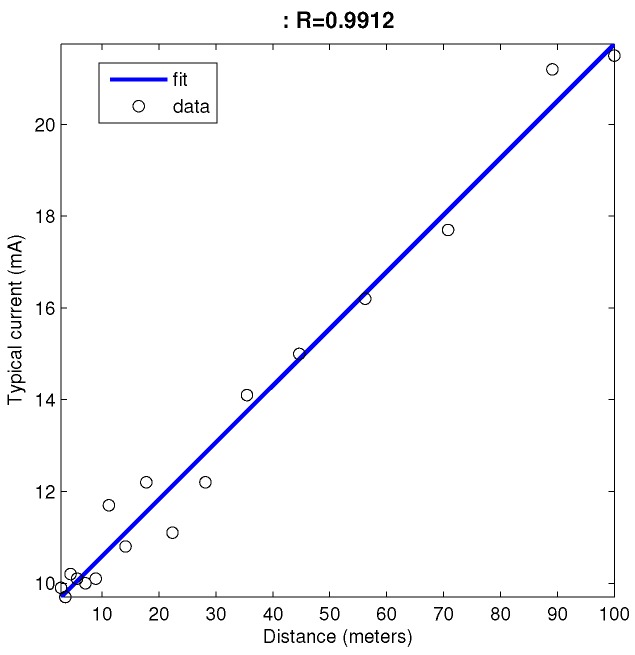
Power consumption vs. distance for CC2500 per packed transmitted.

In this work, we use the simulation environment fully described in [28]. We rely on the freeware Player/Stage environment [42] to run our control system. Player/Stage supports multiple concurrent client connections, so it can simulate every robot in a MRS. The main advantage of this environment is that physical robots can be replaced by simulated ones in a straight way [43]. This feature allowed us to use a few physical robots to fix the heuristic parameters of the proposed algorithm (Table 1) and then use the same software in large networks’ simulation. Our real robots are based on off-the-shelf Hexbug robot toys (Figure 5). Every robot control board circuitry includes an H-bridge for motor control, plus feeding and protection electronics. The board is connected to a EZ430-RF2500 from Texas Instruments Inc. (Dallas, TX, USA), including an MSP430F2274 microcontroller and a CC2500 RF chip. Figure 6 presents the power consumption vs. distance plot for CC2500 RF according to its datasheet [44]. The system uses the Texas SimpliciTI Low Power RF open protocol to operate 2.4-GHz wireless networks. Real robots estimate distances using RSS-based trilateration, but they also include a SHARP infrared range sensor for collision avoidance (robots may avoid collisions with each other using RSS, but they need a sensor to detect dead nodes and other obstacles in the environment).

Robots in simulation always know their location with respect to the others and to the coverage area boundaries. We have only used real robots for parameter setting. In these cases, we relied uniquely on RSS-based trilateration for localization, so localization errors appeared. We did not implement any statistical robotic localization method in our robots for several reasons: (i) the computational load of such processes is high, and our microcontroller memory was mostly dedicated to the communication protocol stack implementation; (ii) our robots are legged, and their body structure does not allow any simple odometry mechanism; (iii) the onboard SHARP sensor has a very limited detection range. Since our deployment algorithm is fully reactive, it would work in real environments despite localization errors. However, if localization precision were required, our robots would need to be replaced to support more complex localization mechanisms.

After the parameters are heuristically fixed (see Section 2.3), a large number of these robots is simulated in Player/Stage to test the behavior of large MWSN during deployment and self-healing. Environments in our simulations are 150 × 150 m${}^{2}$. These environments can be fully covered with 100 of our robots.

### 3.3. Tests Description

The goal of our tests is to show that the proposed behavior-based algorithm: (i) provides good adaptation to the environment; and (ii) provides good performance according to the quality parameters proposed in Section 3.1. Since the proposed algorithm is fit for geographic and hierarchical routing, a secondary goal is to compare the resulting MSWN for both routing strategies in terms of coverage and network life for deployment and self-healing. Hence, in this work, we run four kinds of tests:

- Geographic routing without obstacles
- Geographic routing with obstacles
- Hierarchical routing without obstacles
- Hierarchical routing with obstacles

In all of the tests, we follow the same procedure:

1. Let nodes move until balance, i.e., nodes stop moving (see Section 2).
2. Obtain all relevant quality parameters (see Section 3.1).
3. Determine which nodes would fail first (depending on routed traffic), and move time forwards in the simulation until the most loaded nodes run out of battery (typically, nodes do not fail continuously, but in small groups, depending on how many packets they were routing/rerouting). At this point, forces are not balanced anymore, and remaining living nodes start to move again.
4. Go back to Step 1 until the number of living nodes is lower than 70% of the original number of nodes.

In order to determine in which time instant a given node dies under simulation, its power consumption (current drained from the battery) during network operation has to be estimated. This consumption does not depend only on the transceiver features specified in the datasheet, it is also highly influenced by the medium access control (MAC) strategy, which determines how much time the transceiver spends in each possible state (transmission, reception or sleep) during its operation. In battery-powered wireless sensor networks, a duty-cycling MAC strategy is typically used to allow the transceiver to remain in a low power state most of the time [45]. Our simulation model considers such a strategy. There are two main sources of power consumption: (i) a constant background consumption due to the duty-cycling operation, which is equal for every node in the network; and (ii) the consumption caused by packet transmissions, which depends on how many packets each node is transmitting and how much power is required per packet. If MAC parameters are properly tuned, current drained during duty-cycling operation can be as low as 1% of the current drained when in the receiving state [46]. On the other hand, duty-cycling strategies require each packet to be repeatedly transmitted over a period of time. Hence, transmission becomes more costly and the main source of consumption. Current drained from the battery when transmitting depends on transmission power, which in turn determines transmission range (Figure 6). In geographic routing scenarios, we configure all nodes to transmit their packets at −12 dBm to cover a prefixed range (25 m radius). In hierarchical networks, L0 nodes transmit to L1 nodes, and L1 nodes retransmit to the sink node. In this scenario, we consider that the transmission range is adapted depending on the relative distances among the source and destination nodes, to guarantee that the signal received by the destination is above its sensitivity level. Thus, the current drained from the battery is estimated following the linear regression depicted in Figure 6. In our simulations, we assume an ideal battery with a capacity of 3000 mA·h and a wake-up frequency of 20 Hz for the duty-cycling protocol. To compute the amount of packets routed by each node, we consider a simple data-gathering traffic model, where each node sends a packet to the sink node approximately every 10 s once the network is deployed.

We have added a simplified motor consumption model to the transmission one for our simulations. First, we measured how much current is drained from the battery when the robot is moving (≈100 mA). All of our robots move at constant speed, both in real tests and under simulation. Then, we measured how long it takes for a robot to move 1 m ahead (≈20 s). This provides the motor battery discharge per distance unit (≈0.56 mA·h/m). Finally, in our simulation, this current drain is calculated for each robot depending on its traveled distance. It needs to be noted that this model is over-simplistic, and consumption would probably change in real conditions, especially if the terrain were uneven, robots could accelerate and/or they carried a load. Nevertheless, in all of our tests, we found that power consumption due to motor operation is neglectable with respect to power consumption due to communications, because: (i) the covered area is not too large, so robots do not travel too far even in the worst case scenario (robots in boundary area in environments with obstacles); and (ii) after deployment, robots do not move too often.

## 4. Experiments and Results

All simulations in this section are run in 150 × 150 m${}^{2}$ environments with and without obstacles using 100 nodes. These robots start together at the center of the test environment. Then, they move autonomously until deployment is complete. The sink node does not move: it remains in the center of the deployment area. It does not run out of battery either, because in real tests, it would be connected to a main power source. When nodes die, self-healing is achieved, as proposed in Section 2. All tests are performed for geographic and hierarchical routing.

### 4.1. Topologies after Deployment and Self-Healing

**Figure 7:**
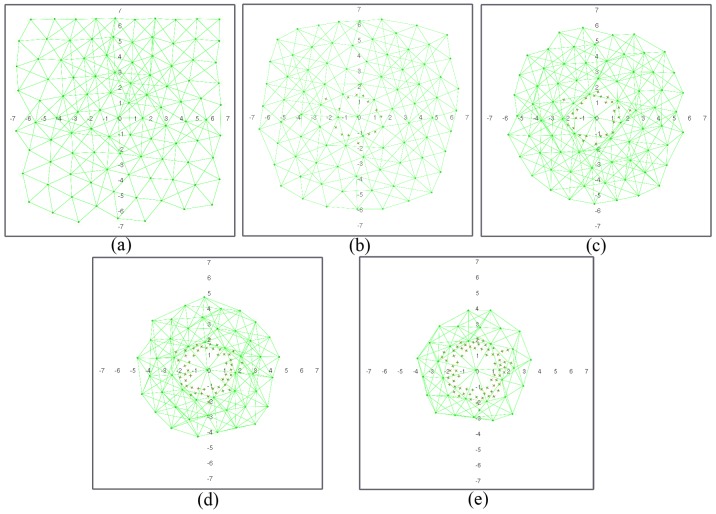
Network configuration when: (**a**) 0%; (**b**) 12.5%; (**c**) 25%; (**d**) 50% and (**e**) 70% of the nodes are dead (figures are scaled 1:10 m).

**Figure 8:**
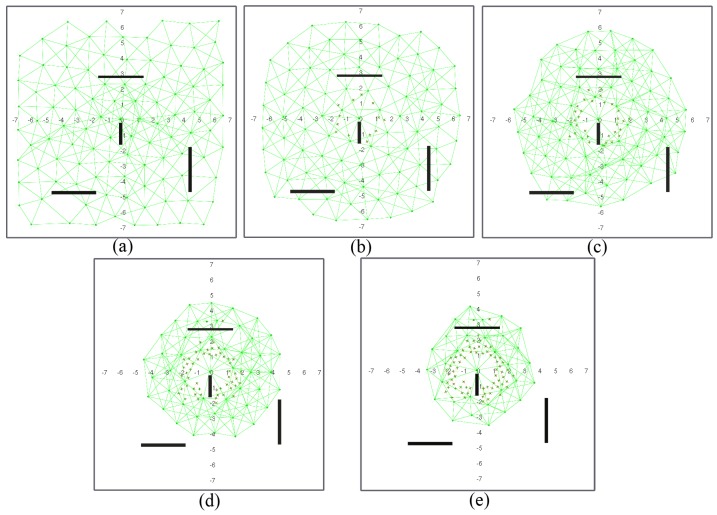
Network configuration when: (**a**) 0%; (**b**) 12.5%; (**c**) 25%; (**d**) 50% and (**e**) 70% of the nodes are dead (figures are scaled 1:10 m).

Figure 7 and Figure 8 show a network with geographic routing in an environment with and without obstacles, respectively, for a decreasing number of living nodes. The proposed algorithm returns a homogeneous distribution of nodes in a grid-like structure. The grid is not fully symmetrical nor equally spaced, because there is no global directive to move one way or another. The initial positions of the nodes after deployment are simply the result of all interacting forces when balance is reached (Figure 7a and Figure 8a). As time passes, nodes start to die, and the grid starts to shrink. As soon as there are not enough nodes to cover the full test environment, remaining living nodes tend to conform a circular structure around the sink node due to its attraction force (Figure 7b–e and Figure 8b–e). It can be observed that nodes close to the sink node tend to die earlier than the rest. This is coherent with a closer neighbor routing algorithm, where nodes close to the destination one reroute most of the traffic. Dead nodes become obstacles. Since $f_{r1}\left(r_{i,j}\right)$ is lower than $f_{r2}\left(r_{i,j}\right)$, living nodes can move closer to dead nodes than to other living nodes. However, at some point, dead nodes might act as a barrier around the sink node for living nodes (Figure 7d,e and Figure 8d,e). In our simulations, this issue was not important because the distance of this barrier to the sink node is lower than the communication range (Figure 7e). If the barrier actually kept outside nodes out of range, it might be necessary to add additional battery-dependent forces to move dying nodes out of the way.

**Figure 9:**
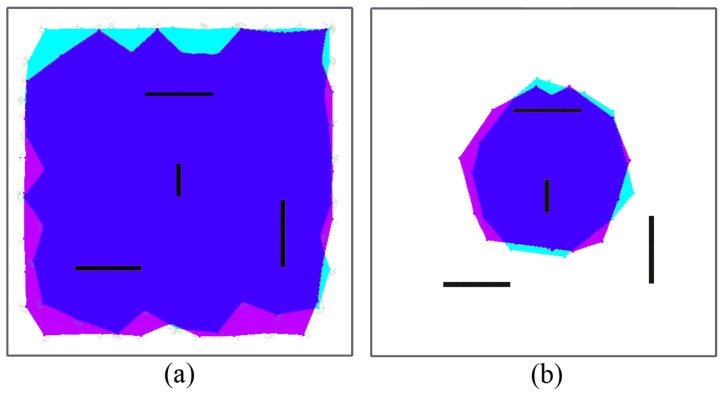
Deployment with geographic routing: area difference with and without obstacles: (**a**) initial; (**b**) final.

One of the main advantages of the proposed algorithm is that it naturally adapts to the structure of the environment. Since nodes operate only on reactive forces, any obstacle in the way simply acts as a repulsor, and nodes seamlessly arrange themselves around it (Figure 8a–e). Obstacles may have some impact on how the network evolves during self-healing, since rearrangement is not optimized at any stage. However, deployment differences in nodes with and without obstacles are not significant. Figure 9 shows the deployment areas after initial balance and after 70% of the nodes are dead in the environment without and with obstacles for the simulations in Figure 7 and Figure 8. We can observe that there are some differences in the boundaries, but nodes are mostly located within the same areas.

**Figure 10:**
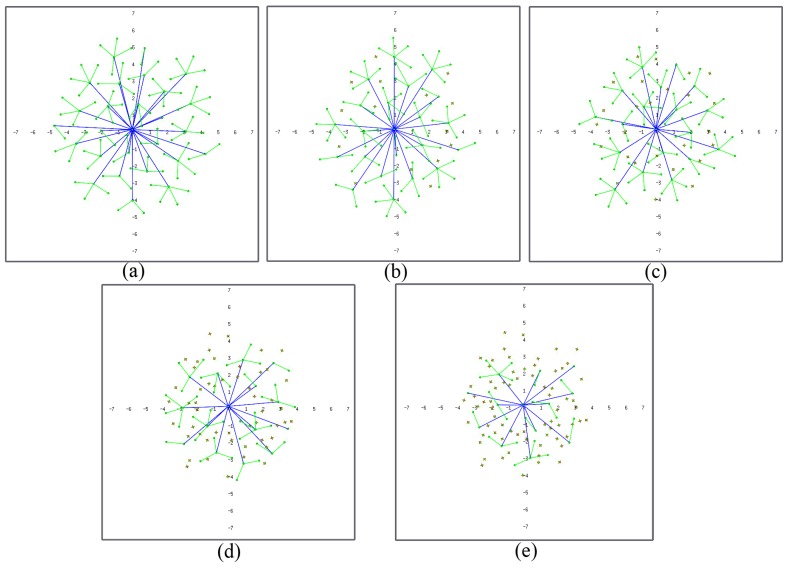
Network configuration when: (**a**) 0%; (**b**) 12.5%; (**c**) 25%; (**d**) 50% and (**e**) 70% of the nodes are dead (figures are scaled 1:10 m).

**Figure 11:**
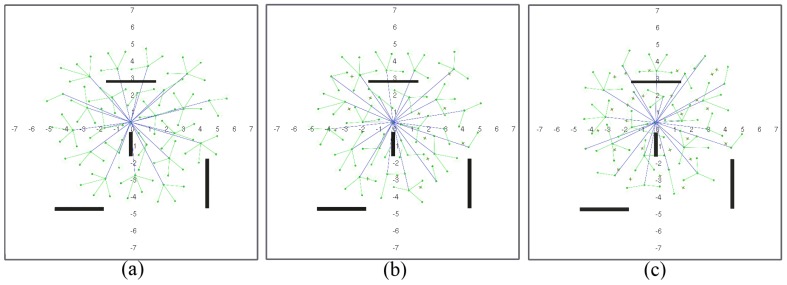

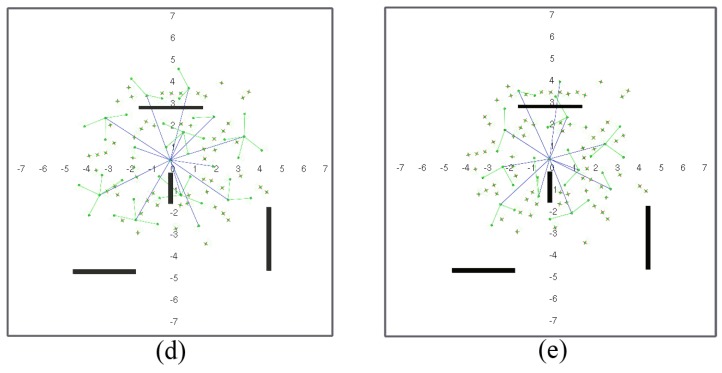
Network configuration when: (**a**) 0%; (**b**) 12.5%; (**c**) 25%; (**d**) 50% and (**e**) 70% of the nodes are dead (figures are scaled 1:10 m).

**Figure 12:**
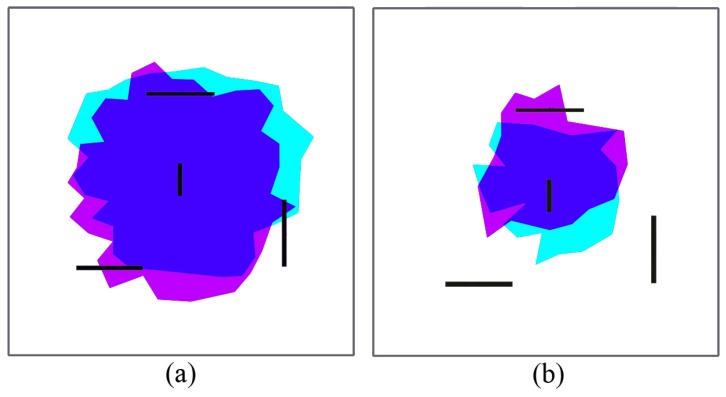
Deployment with hierarchical routing: area difference with and without obstacles: (**a**) initial; (**b**) final.

Figure 10 and Figure 11 show a deployed network with hierarchical routing in the same environment with and without obstacles. If we compare geographic and hierarchical routing network deployment in the same environments (Figure 7 and Figure 8), it is obvious that the coverage area is lower in hierarchical mode. In this case, no node barrier appears around the sink node, because dead nodes are more randomly distributed. In this case, nodes die in the expected order: L1 nodes fail before L0 nodes, and then, they are replaced by role swapping L0 nodes (see Section 2.2, Figure 1). In hierarchical deployment, the proposed algorithm also adapts naturally to environments with obstacles (Figure 11). Since hierarchical networks keep a (minimum) structure, differences in node areas with and without obstacles (Figure 12) are larger than in the previous case (Figure 9), mostly in areas close to obstacles. However, covered areas are basically the same.

### 4.2. Coverage and Network Life

Deployment and self-healing results can be evaluated in terms of the parameters proposed in Section 3.1.

**Figure 13:**
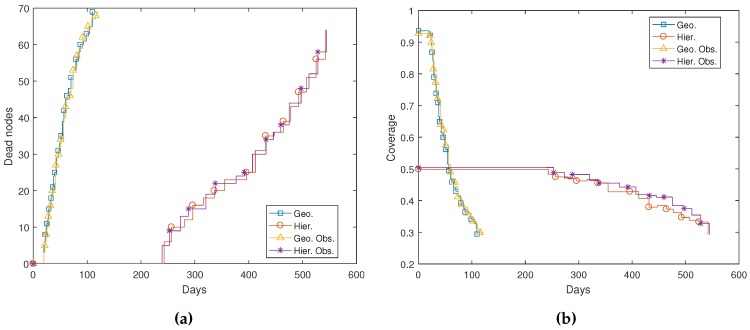
Network evolution in time: (**a**) dead nodes; (**b**) coverage.

The most relevant parameter is coverage. As the number of dead nodes in the MWSN increases, coverage decreases. Figure 13a shows how nodes die in time during the simulations in Figure 7, Figure 8, Figure 10 and Figure 11. Nodes run out of battery mostly depending on the number of packets they route and also on transmission distance (Figure 6). As commented in Section 3, in our closest neighbor routing implementation, all nodes transmit with constant power, specifically at −12 dBm to reach 25 m. In hierarchical routing, packets are sent to the sink node via L1 nodes, and transmission power is adapted to reach the destination node depending on the distance between transmitter and receiver. Nodes usually die in small groups that have been routing similar traffic since the network reached balance.

**Figure 14:**
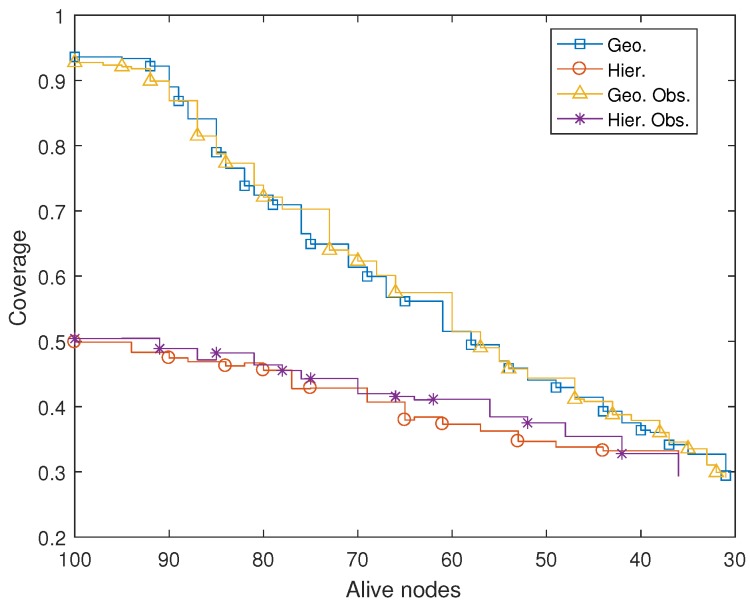
Coverage vs. number of nodes alive in an MWSN deployed with the proposed algorithm.

Figure 13a shows the evolution of dead nodes in time for all simulations in Section 4.1. As commented, results in real conditions would most likely change (e.g., robot battery life, localization errors, etc.), so coverage and network lifetimes would probably be significantly lower. Nevertheless, conclusions extracted from these simulations as a whole are consistent and coherent with theoretical expectations. Closest neighbor routing provokes partial flooding. Hence, the number of packets in the network is much larger in this mode than in hierarchical routing, where an L0 packet is just one hop away from the sink node. Consequently, nodes start to die earlier and faster in geographic routing simulations: around 120 days after initial deployment, 70% of the nodes are dead. In hierarchical routing simulations, nodes do not start to die until approximately Day 250, and their dying rate is lower. Indeed, the network life is extended to almost 600 days. On the other hand, as observed in Section 4.1, coverage in geographic routing scenarios is initially higher. Figure 13b shows how coverage is higher than 90% in these scenarios, both with and without obstacles, whereas it is slightly over 50% for hierarchical routing scenarios. However, coverage decreases much faster in geographic routing simulations: after approximately 60 days, hierarchical networks present a better coverage than non-hierarchical ones. Indeed, they preserve the same coverage until nodes start to die (around Day 250), and afterwards, coverage decreases significantly slower. This effect can be better appreciated in Figure 14, where coverage is plotted against the number of living nodes instead of against time. This was to be expected, since nodes live longer when hierarchical routing is used.

It is important to note that if we changed our routing algorithms, we could improve the performance of geographic routing deployment in our simulations. Similarly, if we change algorithm parameters, like attraction forces, we could improve coverage in hierarchical routing. However, the general conclusions would remain the same: geographic routing deployment with our algorithm provides better coverage, and the hierarchical one provides a longer network life. In this sense, non-hierarchical networks are probably more suited for emergency deployment of MWSN, whereas hierarchical networks are more adequate for long-term deployment.

### 4.3. Node Distribution

**Figure 15:**
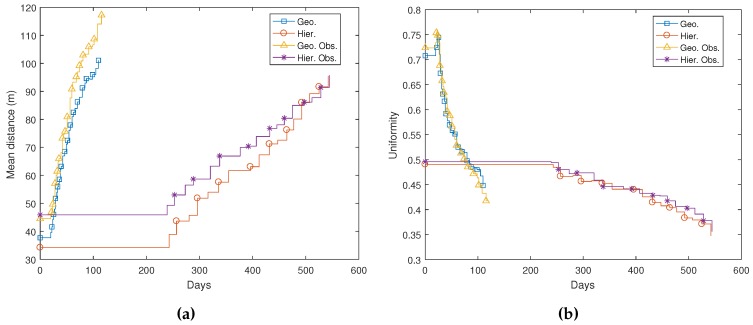
Node distribution evolution in time: (**a**) mean distance per (living) node; (**b**) network uniformity.

Figure 15 shows the evolution in time of parameters related to node distribution. Figure 15a presents the average distance that nodes move in time in all presented simulations. Only living nodes are taken into account to calculate this average. It can be observed that mean distances tend to evolve similarly for non-hierarchical network deployment, although they are initially larger in an environment with obstacles. Nodes move on average 38 m for deployment if there are no obstacles and 44 m for the proposed obstacle layout. Then, when nodes start to die, all remaining nodes adjust their positions, and hence, the mean distance grows steadily. After 100 days, the mean distance is over 100 m for non-hierarchical simulations. Distances also evolve similarly for both hierarchical simulations. After initial deployment, the mean distance is equal to 34 m and 45 m for the environment without and with obstacles. Since nodes last longer in these simulations, they stay in position until Day 250. After that, when nodes start to die, living nodes relocate themselves. In this case, the mean distance grows more slowly, probably because most variations affect only limited areas where L1 nodes died. Nevertheless, the final nodes also reach a mean distance close to 100 m after 70% of the nodes are dead. Figure 15b shows network uniformity. As expected, uniformity is much better in geographic routing simulations, where all nodes have the same roles and, hence, are affected by the same forces. It is interesting to note that when nodes start to die, uniformity punctually grows up to 0.75 for these simulations. This probably happens because after some nodes are dead, the network can extend to its fullest with respect to the simulation area and the allowed communication range (see Equation (4)). Soon afterwards, the number of nodes starts to decrease, and there are not enough nodes to extend over the full simulation area. At this point, uniformity decreases very quickly. In hierarchical routing simulations, uniformity is initially lower, as expected. This is typical for deployment algorithms where nodes tend to conform structures. Nevertheless, like other parameters, including coverage, uniformity decreases much slower in these cases.

### 4.4. Power Consumption

**Figure 16:**
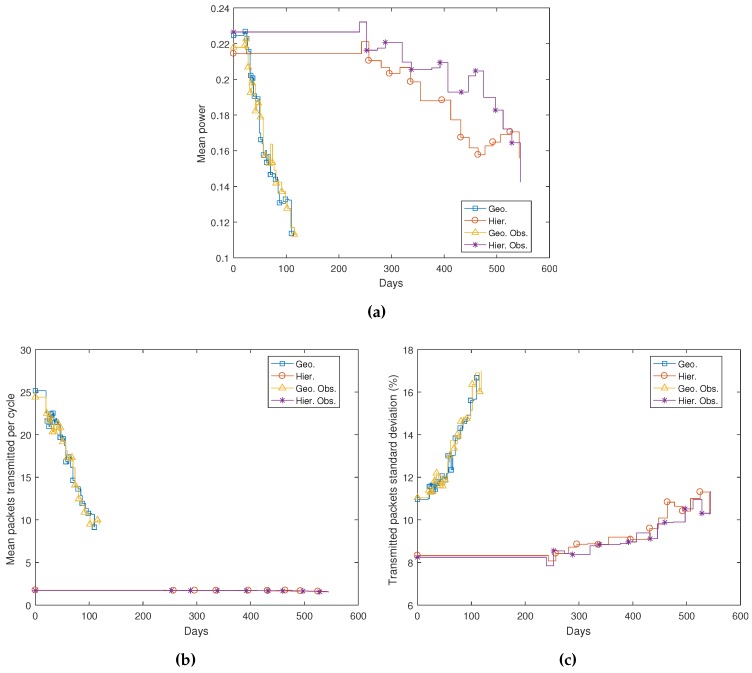
Power evolution in time: (**a**) mean power in the network; (**b**) mean transmitted packets; (**c**) transmitted packets’ standard deviation.

Figure 16a shows mean power in the network (see Equation (6)). Mean power is calculated using only living nodes. As commented, nodes in non-hierarchical simulations transmit at a fixed power, but mean power in the network is reduced as the average number of hops necessary to reach the destination decreases. In hierarchical networks, power depends also on transmission distance. Hence, mean power decreases in time, mostly because living nodes tend to move closer to avoid loss of connectivity. In general, mean power after deployment is higher in simulations with obstacles. However, when nodes start to move closer to each other, their relative locations depend on the obstacle layout, and mean power may grow or decrease punctually depending on the number of living nodes.

Power on the network obviously depends on the number of transmitted packets. This number depends on routing. In closest neighbor routing, a node transmits a packet to all of its neighbors. Even though some of these neighbors, those farther from the sink node than the transmitting node, do not retransmit the packet and a node never transmits the same packet twice, in the worst case scenario, a node close to the sink in an N node MWSN might need to route N-1packets. We can observe in Figure 7 and Figure 8 that geographic routing networks are highly interconnected. Hence, the mean number of packets transmitted over these networks is typically very high, but decreases in time with the number of living nodes (Figure 16b). In hierarchical networks, the mean is very stable because most L1 nodes have approximately the same number of attached L0 nodes, and there is only one retransmission per packet. Transmitted packets’ standard deviation in non-hierarchical networks grows in time, because nodes tend to move closer to each other, and hence, interconnectivity grows; but outside nodes route much less traffic than those closer to the sink node (see Figure 7d,e and Figure 8d,e). In a hierarchical network, this deviation is low and stable until nodes start to die. At this point, the ratio between L1 and L0 nodes grows, and L1 nodes are the ones that contribute to the deviation in these simulations. There are no significant differences in scenarios with and without obstacles in terms of power in the network.

## 5. Conclusions

This work has proposed a reactive behavior-based algorithm for deployment and self-healing in MWSN using autonomous robots. The algorithm is based on locally balancing three forces for each robot: (a) repulsion from nearby obstacles to avoid collisions and remain in the work area; (b) repulsion from nearby nodes to expand the network; (c) attraction to gateway nodes to prevent loss of connectivity. The algorithm is valid for geographic and also for hierarchical routing if we define roles to determine which forces affect a given node. Parameters in our nodes have been heuristically adjusted using a reduced number of physical robots equipped with a radio chip and a range sensor. Afterwards, we have tested the algorithm in large networks using the Player/Stage simulation environment. Tests have been performed in environments with and without obstacles to check how adaptable the proposed algorithm is to environment layout. Results have been evaluated in terms of network life and coverage in time. We have tested the algorithm both for geographic and hierarchical routing, although our routing algorithms are very simple because routing was not the target of this work.

The main advantages of our algorithm with respect to other approaches [4] are the following ones. Our algorithm is valid for multi-node failure. It is fully reactive, so no knowledge about the topology of the full network nor about the layout of the environment is required: nodes estimate their motion in terms of the relative distance to their (one-hop) neighbors and nearby obstacles detected with an onboard short-range sensor. Unlike in game theory-based approaches, no absolute localization information is required. Furthermore, the algorithm does not assume that there is a direct path free of obstacles during movement. The computational load is reduced and distributed among the nodes; this increases resistance to failure. Discrimination between different types of nodes, if necessary, is automatic: nodes reposition themselves depending uniquely on local conditions and their intended role. Additional constraints can simply be added as additional forces in the SPF algorithm. On the other hand, since the proposed algorithm is fully reactive, its performance may be sub-par with respect to algorithms that optimize factors like coverage, average distance traveled by nodes, number of hops to sink, etc. Furthermore, the estimation of node to node distance is based on RSS from nodes in the transmission range. In the real world, returned values would be affected by localization errors. Although collisions are handled by on-board range sensors, relative distances between nodes and, especially, distance to the deployment boundaries would be affected by these errors, as well.

Our main conclusions are the following ones. The proposed algorithm provides good results in terms of deployment and self-healing. Differences between scenarios with or without obstacles are not significant, meaning that the algorithm adapts well to the layout of the environment. Furthermore, it steadily adapts to the loss of nodes in the network, as well. Geographic routing-based networks usually provide better coverage, but their expected life is much lower. This drawback could be mitigated by using more efficient routing algorithms. However, these routing algorithms would also increase signaling traffic, so further study would be needed. Similarly, coverage could be improved in hierarchical routing-based networks if we allowed nodes to travel further from each other, but then transmission power would also need to be increased.

The proposed algorithm is suitable for deployment and self-healing in large, dynamic, unstructured areas, although its performance depends on acceptable node localization. Hierarchical routing is advisable for operation during extended time periods, whereas non-hierarchical routing might be a better alternative to cover large areas quickly, e.g., for emergency deployment of WSN.

Future work will focus on building a larger number of physical robots to run tests in real environments and evaluate the real impact of localization errors and also on testing better routing and topology management strategies to estimate how much the network life could be extended.

## Figures and Tables

**Table 1:** Forces applied to node *i* for every node *j* in its vicinity.

Force	Geographic Routing	Hierarchical Routing			
$f_{r1}\left(r_{i,j}\right)$	$-\frac{0.001}{{\left(r_{i,j}\right)}^{8}}$	$-\frac{0.001}{{\left(r_{i,j}\right)}^{8}}$			
		**L1 vs. SN**	**L1 vs. L1/L0**	**L0 vs. L1**	**L0 vs. L0**
$f_{r2}\left(r_{i,j}\right)$	$-\frac{20}{{\left(r_{i,j}\right)}^{7}}$	$-\frac{20}{{\left(r_{i,SN}\right)}^{2}}$	$-\frac{60}{{\left(r_{i,j}\right)}^{7}}$	$-\frac{10}{{\left(r_{i,j}\right)}^{3}}$	$-\frac{3}{{\left(r_{i,j}\right)}^{8}}$
$f_{c}\left(r_{i,j}\right)$	$\frac{20\cdot N_{fail}}{N\cdot N_{nearSN}}$	$\frac{6}{{\left(r_{i,SN}\right)}^{0.2}}$	—	$\frac{10}{{\left(r_{i,j}\right)}^{0.2}}$	—

## Abbreviations

The following abbreviations are used in this manuscript:

SPF	Social potential fields
MAC	Medium access control
MRS	Multiple robot systems
MWSN	Mobile wireless sensor network
RF	Radio frequency
RSS	Received signal strength
SN	Sink node
WSN	Wireless sensor network
